# Bacterial inclusion bodies function as vehicles for dendritic cell-mediated T cell responses

**DOI:** 10.1038/s41423-019-0298-x

**Published:** 2019-10-08

**Authors:** Sjoerd T. T. Schetters, Wouter S. P. Jong, Laura J. W. Kruijssen, H. Bart van den Berg van Saparoea, Steef Engels, Wendy W. J. Unger, Diane Houben, Joke M. M. den Haan, Joen Luirink, Yvette van Kooyk

**Affiliations:** 1 Department of Molecular Cell Biology and Immunology, Amsterdam UMC, Location VUmc, Amsterdam, The Netherlands; 2 Abera Bioscience AB, Solna, Sweden; 3 Laboratory of Pediatrics, Division of Pediatric Infectious Diseases and Immunology, Erasmus MC-Sophia Children’s Hospital, Rotterdam, The Netherlands; 4 Department of Molecular Cell Biology, Section Molecular Microbiology, Faculty of Science, VU University, Amsterdam, The Netherlands

**Keywords:** Drug delivery, Cellular immunity, Protein vaccines, Antimicrobial responses

Immunogenic antigens for vaccination are often created through the production of recombinant proteins using *Escherichia coli*.^1^ As a side product, aggregates called inclusion bodies (IBs) are formed, containing largely misfolded forms of the overexpressed recombinant protein.^2^ The effect of IBs on the immune system and whether they can be used as effective vaccine products remain unknown. The non-native conformation of proteins upon accumulation in IBs abrogates their use as vaccines aimed at generating high-affinity antibodies.^3^ However, IBs exhibit unique properties, including mechanical and thermal stability due to their intrinsic particulate nature, biocompatibility, high antigenic content, low toxicity and relative resistance to proteases. These characteristics make IBs attractive as an antigenic vaccine formulation for T cell responses to linear epitopes. Whether IBs can trigger adaptive cellular responses initiated via uptake by dendritic cells for presentation to T cells is unknown.

**Fig. 1:**
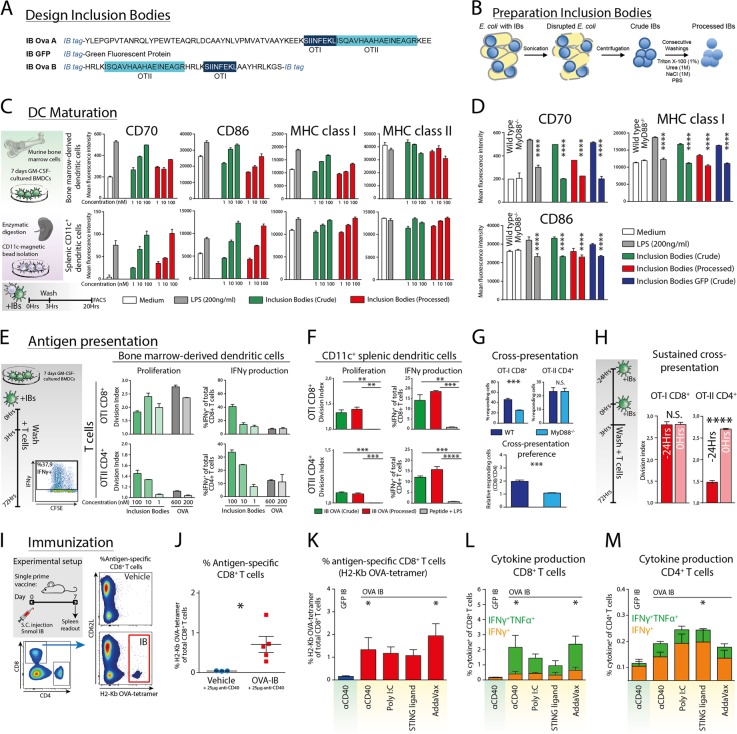
**a** Ova A polypeptide containing the indicated OT-I and OT-II epitopes and GFP were produced in inclusion bodies (IBs) upon N-terminal fusion to IB tag ssTorA(3×).^4^ Ova B polypeptide was produced in IBs upon fusion of a truncated ssTorA(3×) derivative (Jong, Luirink, manuscript in preparation) at both termini. **b** IB production and processing was performed as described^4^ and include sequential washing with Triton X-100 to remove excess membrane material, including LPS, urea to break low-affinity protein interactions and NaCl and high salt to break potential electrostatic protein interactions (processed). **c** Bone marrow-derived dendritic cells (BMDCs, from C57BL/6 WT mice, 7 days GM-CSF cultured^5^) and splenic CD11c^+^ dendritic cells, which were isolated by magnetic beads (MagniSort™/Thermo Fisher; 8802-6861-74) and pulsed with IBs (both crude and processed), show concentration-dependent maturation as measured by FACS. **d** DC maturation induced by IBs in BMDCs from WT or MyD88-deficient mice. **e** DC-mediated antigen presentation was measured as proliferation (CFSE dilution as described previously^10^) and IFNγ production (intracellular staining FACS) by antigen-specific CD8^+^ OT-I and CD4^+^ OT-II transgenic T cells after 3 days of coculture with product-pulsed BMDCs. **f** CD11c^+^ splenic dendritic cells purified by magnetic CD11c-bead isolation were tested for antigen presentation to CD8^+^ and CD4^+^ T cells. **g** OVA^-^IB-pulsed MyD88^−/−^ BMDCs show significantly decreased levels of CD8^+^ T cell activation, but not CD4^+^ T cells, compared to WT BMDCs. **h** BMDCs were cultured with product either 24 or 0 h before coculture with CD8^+^ OT-I and CD4^+^ OT-II transgenic T cells to test the long-term antigen presentation capacity of IB-pulsed BMDCs. The effect of 24 h of processing had little effect on the cross-presentation of IB-pulsed BMDCs to CD8^+^ T cells but highly reduced CD4^+^ T cell activation. **i** C57BL/6 WT mice were subcutaneously immunized with 5 nmol OVA-IB with 25 µg agonistic CD40 antibody. After 7 days, the mice were sacrificed, and the splenocytes were analyzed by flow cytometry to detect OVA antigen-specific CD8^+^ T cells by H2-Kb OVA-tetramer staining. Representative experiments with both OVA-A and OVA-B IBs. **j** OVA-IBs induced de novo antigen-specific CD8^+^ T cell responses in vivo. **k** Mice (*N* = 9 per group) were immunized with 5 nmol OVA-B IBs in combination with a variety of adjuvants, and antigen-specific CD8^+^ T cells in the splenocytes were measured by FACS using H2-Kb OVA-tetramer staining. **l**. The splenocytes from immunized mice were restimulated for 6 h with 10 µg/ml OVA-derived SIINFEKL short peptide in the presence of brefeldin A, and IFNγ/TNFα production by CD8^+^ T cells was measured by intracellular FACS staining. **m** Similarly, CD4^+^ T cells were restimulated with the OVA-derived ISQAVHAAHAEINEAGR short peptide for 48 h, and IFNγ was measured by intracellular FACS staining. All data are representative of at least two individual experiments. In vitro experiments were performed as biological triplicates, and OVA-A IBs were used. In vivo experiments were performed using both OVA-A and OVA-B IBs, yielding comparable results. Graphs show the mean ± SEM. Statistics applied by GraphPad PRISM 7.0; D/H/J unpaired Fisher’s *t*-test, F/G/K/L/M one-way ANOVA with Tukey’s post hoc comparison; *p < 0.05, **p < 0.01, ***p < 0.001, ***p < 0.0001

We generated IBs containing the ovalbumin (OVA)-derived OT-I and OT-II epitopes using the TorA signal sequence in *E. coli* (Fig. 1a), as previously described.^4^ Since dendritic cells (DCs) are the primary cell type responsible for the activation of T cells, we first analyzed the inherent immunogenic capacity of IBs directly on these cells. IBs were derived from *E. coli* by cell disruption and centrifugation (Crude), as well as additional processing by sequential washing (Processed; Fig. 1b). Bone marrow-derived dendritic cells (BMDCs) were cultured from the bone marrow of C57Bl/6 wild-type mice, as previously described.^5^ Immunogenic maturation of BMDCs was measured by the expression of the costimulatory markers CD70, CD80, and CD86, and the MHC Class I and II complexes using flow cytometry. Both unprocessed (Crude) and processed IBs induced the expression of CD70, CD80, CD86, and MHC class I in a concentration-dependent manner, whereas MHC class II was downregulated by IBs at higher (100 nM) concentrations, as was observed with LPS (Fig. 1c). Additionally, splenic CD11c^+^ DCs showed similar concentration-dependent maturation by IBs as BMDCs. Although IBs are particulate aggregates that are structurally resistant to detoxification methods, such as Triton-mediated phase separation (removing free LPS), IBs are rarely endotoxin free.^6^ Since endotoxins such as LPS are sensed by TLR4 on innate immune cells, resulting in MyD88-mediated NFκB activation,^7^ we tested the effect of IB-induced maturation on MyD88-deficient BMDCs. Interestingly, loss of MyD88 in BMDCs completely abrogated IB-induced maturation (Fig. 1d), suggesting that intact TLR signaling is required for IBs to induce maturation in DCs. Although free LPS is effectively absent in processed IBs, the maturation of BMDCs by processed IBs remained MyD88-dependent and was independent of antigen content since both OVA-containing (Fig. 1d; green and red) and GFP-containing IBs (Blue; Fig. 1d) induced MyD88-dependent DC maturation. In summary, IBs show an inherent capacity to induce DC maturation that is dependent on the presence of MyD88, downstream of TLR signaling.

IBs have strong DC maturation capability, indicating their potential as vaccine candidates, since DC maturation is required for the effective priming of naïve CD8^+^ and CD4^+^ T cells. However, it is unknown whether DCs can internalize and process IB-included antigens for antigen presentation. Therefore, we tested the capacity of processed IBs to induce antigen presentation by DCs to CD8^+^ and CD4^+^ T cells. BMDCs were cultured in the presence of OVA-IBs, engineered to include OVA-derived OT-I and OT-II epitopes, or ovalbumin protein for 3 h, extensively washed and cocultured with antigen-specific CFSE-labeled OT-I CD8^+^ or CD4^+^ O-TII transgenic T cells for 3 days (Fig. 1e). We observed concentration-dependent proliferation and production of IFNγ by both antigen-specific OT-I CD8^+^ T cells and OT-II CD4^+^ T cells after coculture with IB-pulsed BMDCs (Fig. 1e). Purified splenic CD11c^+^ DCs loaded with OVA-IBs were also capable of inducing antigen-specific proliferation and IFNγ production in both CD8^+^ and CD4^+^ T cells (Fig. 1f). Proliferation and IFNγ production induced by IBs were significantly higher than those induced by equal amounts of synthetic long peptide containing OT-I and OT-II sequences. Hence, both cultured BMDCs and isolated CD11c^+^ splenic DCs take up, process and present IB-derived antigens to CD8^+^ and CD4^+^ T cells. The uptake, processing and presentation of exogenous antigen on MHC class I to CD8^+^ T cells is a process called cross-presentation.^8^ TLR4 engagement, MyD88 signaling and DC maturation by LPS have previously been shown to enhance cross-presentation.^9^ Therefore, we tested the capacity of BMDCs from MyD88-deficient mice to cross-present IB-derived antigen to CD8^+^ T cells. MyD88-deficient BMDCs showed a reduction in CD8^+^ T cell activation, while CD4^+^ T cell activation was unaffected (Fig. 1g). Quantification of the preference for cross-presentation (calculated percentage of initially responded CD8^+^ T cells divided by CD4^+^ T cells) showed a significant decrease in cross-presentation when MyD88 signaling was lost. Hence, intact TLR-MyD88 signaling is required for optimal cross-presentation of IB-derived antigens. Lastly, we hypothesized that the increase in antigen presentation of IB-derived antigens was due to intracellular depot-formation and slow release of antigen from internalized IBs. To test this, we incubated BMDCs with IBs (for 3 h and with extensive post-incubation washing) 24 h before T cell coculture or without the 24-h preincubation (Fig. 1h). Interestingly, no reduction of CD8^+^ T cell activation induced by IBs was observed when DCs were preincubated with IBs, suggesting intact and continued cross-presentation (Fig. 1h). In contrast, CD4^+^ T cell activation was significantly decreased with preincubation for 24 h, suggesting that MHC class II-mediated antigen presentation to CD4^+^ T cells occurs mostly within the first 24 h after IB incubation.

Having verified the capacity of IBs to induce DC-mediated antigen presentation to T cells, we next investigated the capacity of IBs to generate de novo cellular responses in vivo. First, we subcutaneously injected OVA-IBs with agonistic CD40 antibody in mice, and after 7 days, measured the percentage of ovalbumin-specific CD8^+^ T cells in splenocytes by flow cytometry using an H2-Kb OVA-tetramer (Fig. 1i). A significant induction in antigen-specific CD8^+^ T cells was observed in the splenocytes of immunized mice after 7 days (Fig. 1j). IBs without any additional adjuvants did not induce detectable antigen-specific CD8^+^ T cells (data not shown). To test whether the induction of cellular responses could be further optimized, we vaccinated mice with IBs combined with a selection of adjuvants known to boost CD8^+^ T cell responses, including agonistic CD40, Poly I:C, AddaVax (MF59) and STING ligand (DMXAA). AddaVax and agonistic CD40 induced the highest frequency of CD8^+^ T cells as measured by tetramer staining (Fig. 1k). When IBs were combined with agonistic CD40 or AddaVax, the highest frequency of CD8^+^ T cells producing the effector cytokines IFNγ and TNFα after peptide restimulation was found (Fig. 1l). Surprisingly, the frequency of CD4^+^ T cells producing IFNγ upon peptide restimulation showed an inverse correlation to the CD8^+^ T cell responses (Fig. 1m). Hence, the adjuvants may determine the magnitude of the CD8^+^ or CD4^+^ T cell response to IBs. In summary, we experimentally showed the potency of IBs to be used as a vaccine to induce both strong antigen-specific CD8^+^ and CD4^+^ T cell responses.
